# Nanoparticle-Based Biosensing Assay for Universally Accessible Low-Cost TB Detection with Comparable Sensitivity as Culture

**DOI:** 10.3390/diagnostics9040222

**Published:** 2019-12-13

**Authors:** Ruben Kenny Briceno, Shane Ryan Sergent, Santiago Moises Benites, Evangelyn C. Alocilja

**Affiliations:** 1 Instituto de Investigacion en Ciencia y Tecnologia, Universidad Cesar Vallejo, Trujillo 13100, Peru; rubskenny@gmail.com (R.K.B.); shane.sergent@gmail.com (S.R.S.); sbenites@ucv.edu.pe (S.M.B.); 2 Hospital Victor Lazarte Echegaray, Trujillo 13100, Peru; 3 Institute for Global Health, Michigan State University, East Lansing, MI 48824, USA; 4 Kingman Regional Medical Center, Kingman, AZ 86409, USA; 5 Global Alliance for Rapid Diagnostics, Michigan State University, East Lansing, MI 48824, USA; 6 Biosystems and Agricultural Engineering, Michigan State University, East Lansing, MI 48824, USA

**Keywords:** nanoparticles, *Mycobacterium tuberculosis*, biosensing assay, smear microcopy, acid-fast bacilli

## Abstract

Tuberculosis (TB) is the leading cause of death globally, surpassing HIV. Furthermore, multidrug-resistant and extensively drug-resistant TB have become global public health threats. Care of TB patients starts with quality, accessible, and affordable diagnosis. The study presents a novel technique called nanoparticle-based colorimetric biosensing assay (NCBA) based on the principles of magnetically activated cell enrichment. A total of 1108 sputum samples were subjected to sputum smear microscopy (SSM), NCBA, and standard culture. SSM and NCBA were completed in 20 min; culture was completed in 8 weeks. Results show that NCBA has matching sensitivity of 100.0% and specificity of 99.7% compared to the gold standard culture method at a cost of $0.50/test based on Peruvian conditions. Sputum smear microscopy has 63.87% sensitivity compared to culture. NCBA has the potential of being used in local health clinics as it only requires a microscope that is widely available in many rural areas. Because NCBA could detect low levels of bacterial load comparable to culture, it could be used for rapid and early TB-onset detection. The gain in time is critical as TB is airborne and highly infectious, minimizing contact exposure. Early detection could lead to early treatment, while the patient’s immune system is still high. The low cost makes NCBA affordable and accessible to those who need them the most.

## 1. Introduction

Annually, about 1.6 million die from tuberculosis (TB) [1], with 9.4 million new cases around the world [2]. TB is the leading cause of death globally, surpassing HIV since 2014 [3,4]. Because TB is highly infectious, each person with undiagnosed and untreated smear-positive TB is estimated to cause 10–14 infections per year, where about 10% would eventually become a new case of TB [5,6]. Furthermore, multidrug-resistant and extensively drug-resistant TB (MDR/XDR-TB) have become global public health threats. There are many TB patients who have no access to health care facilities or proper diagnosis and treatment [4]. In 2018, Heads of State at the United Nations called for action to end TB^3^. The current annual rate of decline in TB incidence is around 1% to 2%, however, the rate would need to be 4% to 5% by 2020 and over 10% by 2025 in order to achieve the goal of ending the epidemic by 2030 [4]. Substantial annual reductions in TB incidence and the number of TB deaths will be necessary to meet the U.N. Sustainable Development Goals and WHO End TB Strategy targets for 2030 and 2035 [7].

### 1.1. Constraints of Current Methods

Care of TB patients starts with quality, accessible, and affordable diagnosis. The majority of the TB patients live in poor conditions and in geographically remote areas [8]. The culture method is still the “gold standard” for identifying *Mycobacterium tuberculosis* (Mtb) in clinical samples, however, this method takes about 8 weeks to be completed. For decades, TB diagnosis has relied on direct (unconcentrated) sputum smear microscopy (SSM), which is the first microbial analysis both for TB diagnosis and assessment of patient infectiousness in many countries [9]. SSM is fast, inexpensive, easy to perform, and specific for Mtb in high incidence areas [10,11,12]. It does not require complex laboratory equipment and is, therefore, very suitable for low-resource settings and in various populations with different socio-economic conditions [11,12]. However, it has significant limitations in its performance. SSM’s sensitivity is only about 25%–65% compared to culture, with a detection limit of about 5000–10,000 colony-forming units per milliliter (CFU/mL) [10,13]. In a retrospective study comparing culture, SSM, and Xpert MTB/RIF system involving hundreds of specimens, SSM had 54% sensitivity for respiratory samples and 50% for non-respiratory samples [14]. Furthermore, smear sensitivity varies with the type of lesion, type and number of specimens, mycobacterial species, staining technique, and the alertness and persistence of the microscopist [13]. In a recent survey, Kik et al. [15] showed that the 22 high-burden countries (HBCs) conducted 77.6 million sputum smears in 2012 valued at US$137 million in 42,827 microscopy centers [15]. Of these, 61% were performed in the BRICS countries (Brazil, Russian Federation, India, China and South Africa) [15]. On average, 79% of the smears were performed for initial diagnosis in these countries. When converted to 2012 US$, the unit cost for a smear, including materials, labor, and overhead expenses, was US$1.77 [15]. Studies have shown that the sensitivity of SSM improved significantly when specimens are subjected to liquefaction, followed by the concentration of the mycobacteria by overnight sedimentation or centrifugation [10,16,17,18,19,20]. However, the increased sensitivity provided by these processing methods may not be sufficient to offset their increased cost, complexity, and potential biohazards.

Culture is the gold standard, but it is more expensive, and results take weeks [12]. Several molecular techniques have been commercialized for detecting Mtb and mutations in *rpoB* and *katG* genes that cause resistance to Rifampicin and Isoniazid, such as Cepheid’s Xpert MTB/RIF system and line probe assays [14,21,22,23,24,25]. In many studies, the Xpert system was shown to have a sensitivity of 96.8% and a specificity of 99.3% compared to culture as the reference standard [14]. However, they are not necessarily accessible or affordable to those who need them the most [26]. For example, if the Xpert MTB/RIF assay (cartridge price of US$9.98) were to be used for all people with presumed TB, the cost would exceed 80% of the total TB spending in countries such as India, Bangladesh, Indonesia, and Pakistan [27].

An important aspect of TB is the huge financial burden it places on patients and their families, not only for treatment costs but also associated costs, such as that TB patients are required to take a leave of absence from work leading to the risk of impoverishment [4]. Tanimura et al. reported that, on average, 20% of the total cost was due to direct medical costs, 20% to direct non-medical costs, and 60% to income loss [28]. On average, the total cost was equivalent to 58% of reported annual individual and 39% of reported household income [28]. The cost as a percentage of income was particularly high among poor people and those with multidrug-resistant TB [28].

### 1.2. Novelty of the Paper

Accurate, rapid, and cost-effective diagnostic tests are crucial to reducing TB’s unacceptably high infection and mortality rates, especially for a disease that is treatable [29]. Thus, this paper presents a low-cost biosensing assay that integrates modern advances in nanoparticle science and glyco-chemistry, resulting in sensitivity matching the performance of standard culture. The nanoparticle-based colorimetric biosensing assay (NCBA) is based on the concept of magnetically activated cell enrichment (MACE) technique that we have developed, where Mtb cells are isolated and enriched by applying a magnetic field to activate nanoparticle-bound Mtb cells, without using any expensive antibodies and energy-consuming centrifuge instrument and without employing the time-consuming growth of Mtb. Early detection can lead to early treatment while the patient’s immune system is still strong, helping to better treatment response and faster recovery. Affordable and rapid TB detection techniques increase access in poor communities. This holistic strategy will help achieve WHO’s End TB Strategy (2016–2035) and the Sustainable Development Goals (2016–2030) [30].

This study was conducted in Peru, where 3.2 million SSM were performed in 2017 [31]. Peru has the second-highest TB incidence and has the highest MDR-TB in the Americas [32]. Therefore, aggressive measures to detect TB early and widely is an appropriate strategy.

Our previous studies on NCBA for TB detection included quantitative capture efficiency and concentration factor in sputum samples by Gordillo et al. [33] and Bhusal et al. [34]. In Gordillo’s study, we determined that NCBA could concentrate acid-fast bacilli (AFB) Mtb by 47% compared to SSM. Results also showed that NCBA improved the AFB grade from “1+” (in SSM) to “2+” (in NCBA), which would be extremely helpful in detecting paucibacillary TB cases. Bhusal et al. compared the NCBA technique with the WHO-endorsed GeneXpert MTB/RIF system as the standard [34]. In Bhusal’s study, NCBA had 100.0% concordance with the Xpert system in 500 samples demonstrating comparable performance, yet the NCBA required a much shorter time (20 min) and cost pennies without the need for expensive instrumentation or laboratory facility.

### 1.3. Novelty of the MACE Technique

Cell enrichment through glycan-coated magnetic nanoparticles is a novel concept in TB detection. Mtb are isolated and purified from the complex matrix resulting in a much “cleaner” sample and then concentrated through volume reduction without using a centrifuge. Since TB is an airborne disease, centrifugation of TB samples is dangerous due to potential aerosol exposure to medical workers. Whereas with magnetic activation, there is no aerosolization; isolation and concentration are achieved simply by using an inexpensive magnet. The as-prepared glycan-coated magnetic nanoparticles (GMNP) have superparamagnetic properties—the nanoparticles become magnetic only in the presence of a magnetic field—thus they remain colloidal and suspended in solution due to both steric and coulombic repulsions. Their nanoscale size results in their higher effective surface area, lower sedimentation rate, and minimal precipitation from gravitational forces [35]. The glycan-coating facilitates attachment on the bacterial cell wall through the carbohydrate-binding protein sites on the bacterial surface, providing generalized microbial specificity to the GMNP-cell interaction without using expensive antibodies. Due to the superparamagnetic properties of GMNP, the GMNP-Mtb complex also becomes superparamagnetic and, therefore, can be manipulated using an external magnetic field.

## 2. Materials and Methods 

### 2.1. Chemicals and Reagents

Glycan-coated magnetic nanoparticles (GMNPs) were prepared by synthesizing Fe_3_O_4_ using ferric chloride hexahydrate (FeCl_3_.6H_2_O) as a precursor in a mixture of ethylene glycol (as reducing agent) and sodium acetate (as porogen). Chitosan was polymerized to surface-modify the iron oxide nanoparticles. GMNP solution at 5 mg/mL was prepared for use in the studies. Carbol fuchsin (0.3% primary stain) was prepared by dissolving 50 g phenol in 100 mL of 90% ethanol and then adding 3 g of basic fuchsin into the mixture. Distilled water was added to bring the total volume to 1 L. The decolorization solution was a mixture of 95% purity ethanol and hydrochloric acid (HCl). The counterstain was 0.3% methylene blue.

### 2.2. Instrumentation

Instruments used in the study included a bright field microscope (Motic 3.0 MP, USA) and a three-dimensional (3-D) printed magnetic rack containing Neodymium magnets.

### 2.3. Clinical Samples

**Figure 1:**
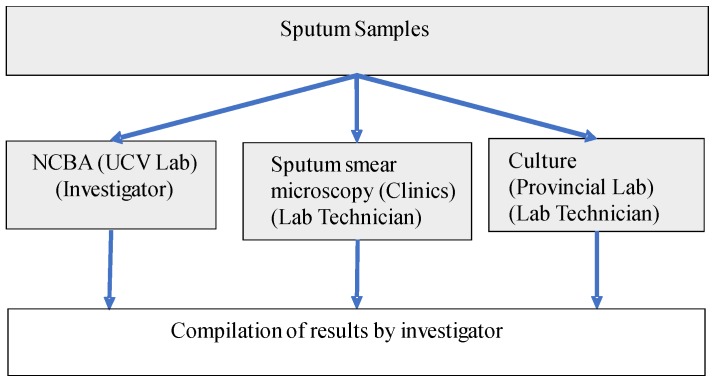
Schematic diagram for handling and processing the sputum samples.

A total of 1108 sputum samples from patients symptomatic of respiratory TB [36] were used in this study. These samples were collected in sterile screw-capped containers from patients who went to 9 health clinics in the La Libertad region of Peru. There were no samples from contacts or asymptomatic patients of TB or unrelated to TB. All samples were subjected to the 3 TB detection methods: SSM, NCBA, and culture as shown in Figure 1. SSM was conducted at the health clinics. Aliquots of the samples were sent to the Provincial Laboratory in Trujillo City for standard culture on Lowenstein Jensen medium. Aliquots of the samples were also sent to the Microbiology Lab at the Universidad Cesar Vallejo (UCV) for NCBA testing. All patient-related information was removed and the samples were decoded and randomized to non-linked codes prior to sending the samples to UCV. Samples were stored at 4 °C upon receipt and processed immediately or within 24 h.

### 2.4. Sputum Smear Microscopy (SSM) by Ziehl–Neelsen Staining

A smear was prepared from the sputum sample following the WHO standard protocol for Ziehl–Neelsen staining (ZN) technique [37]. Briefly, 20 µL of the sputum sample was placed on a microscope slide and heat-fixed by passing flame from a Bunsen burner under the slide. The slide was then placed on a staining rack and 0.3% carbol-fuchsin was poured over the smear. The underside of the slide was gently heated by passing a flame under the rack until fumes appeared, repeated 3 times. After cooling (~2 min), the smear was rinsed with distilled water until no color appeared in the effluent, followed by washing with a mixture of 95% purity ethanol and 3% HCl until the smear appeared light pink. The smear was washed with distilled water and then 0.3% methylene blue was added to cover the smear. Distilled water was used to wash off the counter stain and then the smear was air-dried. Once ready, the smear was examined under a bright field microscope using a 100× oil immersion objective to observe the presence of red-colored acid-fast bacilli (AFB). SSM was completed in about 20 min.

### 2.5. Standard Culture by the Lowenstein-Jensen Method

Aliquots of all 1108 samples were forwarded to the Provincial Laboratory in Trujillo City for culture using the Lowenstein-Jensen (LJ) medium following WHO standard protocols [37]. Briefly, a slant was inoculated with 200 μL of decontaminated sputum specimen using a sterile graduated disposable pipette in a biosafety cabinet. The inoculum was spread evenly over the entire surface of the medium. The tube was left in a slanted position with the cap loosened until the inoculum was absorbed (about a week), then the cap was tightened, and the tube was incubated in an upright position at 37 °C (± 1 °C). The culture was examined weekly for 8 weeks. To observe growth, a strong direct light from an anglepoise lamp was shone onto the slant surface until buff-colored, dry colonies were observed.

### 2.6. Nanoparticle-Based Colorimetric Biosensing Assay (NCBA)

Decoded aliquots of all 1108 samples were sent to UCV and subjected to NCBA. Briefly, 1 mL of sample was added into a 2 mL sterile microcentrifuge tube containing 0.25 mL of 5 mg/mL GMNP solution. GMNP and sputum were mixed by shaking the closed tube and then allowed to incubate for 5 min at room temperature. The tube was then placed in a magnetic rack to separate the magnetic GMNP-cells and the supernatant was discarded as biohazard waste. The isolated GMNP-cells were re-suspended in 0.5 mL of 0.01 M PBS and mixed. About 20 uL of the sample was transferred onto a microscope slide and subjected to the Ziehl–Neelsen staining, as described above. Once ready, the smear was viewed under a bright-field microscope (Motic 3.0 MP, USA) using 100× oil immersion objective to observe the presence of rod-shaped red clumps surrounded by brown nanoparticles. NCBA was completed in about 20 min.

### 2.7. Mathematical Modeling of the Mtb Population

In order to understand the growth dynamics of Mtb, a logistic growth model was used as described by the following differential equation:

(1) $$\frac{dN}{dt}=rN\left(1-\frac{N}{K}\;\right)$$

which can be integrated to give the following solution:

(2) $$N\left(t\right)=\frac{K}{1+\left(\frac{K}{No}-1\right)e^{-rt}}$$

where N(t) is the population at any time t, No is the initial population at time zero, K is the carrying capacity, r is the growth rate, and t is time.

### 2.8. GMNP-Cell Interaction for Transmission Electron Microscope (TEM) Imaging

In order to understand the interaction between GMNP and Mtb, a supplemental study was conducted using *Mycobacterium smegmatis* (Msm) as a surrogate for Mtb due to biosafety considerations. Msm shares a similar cell wall structure with Mtb, both are acid-fast species, and both can be stained using the ZN technique [38,39]. Msm culture was grown in MiddleBrook 7H9-ADC broth and incubated until the optical density reached 0.6 at 600 nm (about log phase). Serial dilutions of the Msm bacteria were prepared and spiked into prepared artificial sputum, followed by incubation for 5 min at room temperature along with manual shaking. The GMNP-Msm were magnetically separated using a simple magnetic rack and the supernatant was removed. GMNP-Msm were washed twice and re-suspended in 0.5 mL of 0.01 M PBS. GMNP-Msm, along with pure bacterial dilutions, were plated on a MiddleBrook 7H10-ADC agar and incubated for 3–5 days to allow the growth of Msm cells. Colonies were counted and converted to CFU/mL. Msm and GMNP-Msm were visualized using a JEOL 100 CX transmission electron microscopy (TEM) at the MSU Center for Advanced Microscopy with a magnification range of 5000 to 80,000×.

### 2.9. Ethics

Ethical approval was obtained from the Research Committee at the Universidad Cesar Vallejo (UCV), Trujillo, Peru, under the project entitled “Eficacia diagnostica del Biosensor de Nanoparticulas Magneticas (BNPM) para Tuberculosis Pulmonar en Pacientes que acuden a Hospitales Distritales de Trujillo, 2017”. Ethical approval was also obtained from the Research Committee at the Gerencia Regional de Salud La Libertad, Trujillo, Peru. Aliquot sputum samples that were sent to UCV were used only for the purpose of this study, and the remaining samples were disposed of according to biosafety guidelines.

## 3. Results

### 3.1. GMNP

**Figure 2:**
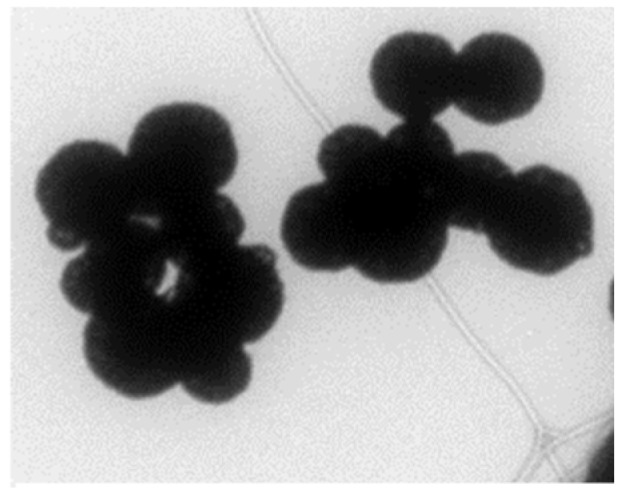
TEM image of clustered glycan-coated magnetic nanoparticles (GMNP).

Synthesized GMNP was characterized by morphology, size, conductivity, and surface charge. Figure 2 shows a transmission electron microscope (TEM) image of GMNP showing spherical shape. Using a laser microscope, it was determined that the average size was 159 ± 88 nm. Using a ZetaSizer, the average conductivity of GMNP was 0.06 ± 0.04 mS/cm and its surface was positively charged. It was highly soluble in water and stored well at room temperature (25–45 °C).

### 3.2. GMNP-Cells

**Figure 3:**
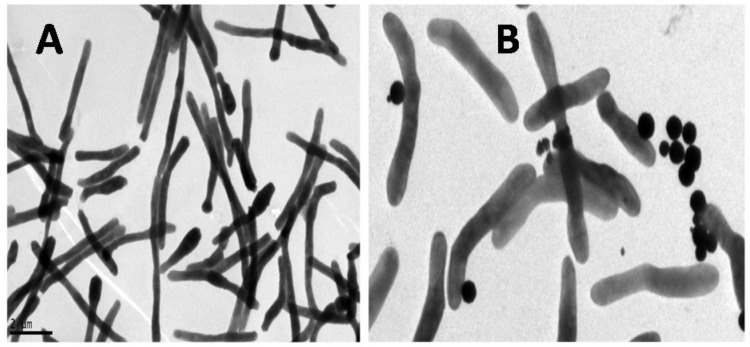
TEM image of *M. smegmatis* (**A**) and GMNP binding to specific sites on *M. smegmatis* cell surface (B).

Figure 3 shows the TEM images of *M. smegmatis* (without GMNP) (A) and with GMNP forming GMNP-cells (B). As shown, the GMNP are attached to specific sites on the bacterial surface.

### 3.3. Clinical Samples

Table 1 shows the distribution of the samples from the 9 local health clinics. Clinic 3 has the largest number of samples at 19%, followed by Clinic 9 at 17% out of the total 1108 samples. Clinic 5 has the lowest number of samples at 5%.

### 3.4. NCBA vs. SSM vs. Culture

Table 1 also shows the results for each local clinic using the three methods: SSM, NCBA, and culture. Clinic 3 has the highest number of positive samples by SSM (36), NCBA (50), and culture (50), followed by clinic 6 (22, 36, 36), clinic 1 (15, 18, 17), and clinic 8 (13, 25, 25). On the other hand, clinic 9 and clinic 7 have the lowest number of positive samples for all three methods (5, 10, 9). Of the total 1108 samples, 122 were positive by SSM, 194 by NCBA, and 191 by culture.

**Figure 4:**
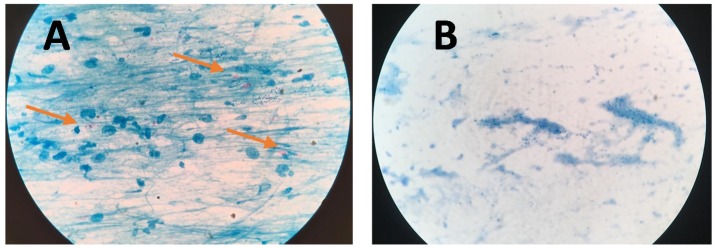
Microscope images of sputum smear microscopy (SSM) for (**A**) TB positive sample showing dispersed red bacilli (pointed by arrows) and (**B**) TB negative samples.

**Figure 5:**
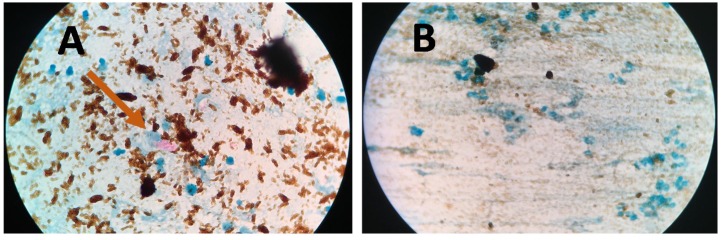
Microscope images of NCBA for (**A**) TB positive sample showing clumped red bacilli (pointed by arrow) and (**B**) TB negative sample showing brown nanoparticles.

**Figure 6:**
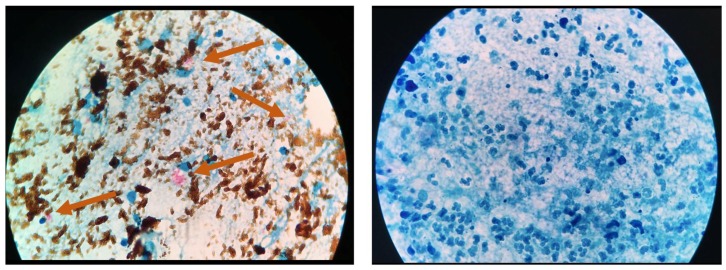
Microscope images of TB positive sample using (**A**) nanoparticle-based colorimetric biosensing assay (NCBA) showing clumped red bacilli (pointed by arrows) and (**B**) SSM of the same sample (negative reading).

Figure 4 presents bright-field microscope images of acid-fast bacilli (AFB) of Mtb using SSM in TB positive sample showing scant and highly dispersed red AFB (Figure 4A) and in TB negative sample showing no red AFB (Figure 4B). Figure 5 presents bright-field microscope images of AFB using NCBA. Figure 5A shows red clumps of AFB in TB positive samples with distinct characteristics: clumped red rods surrounded by brown nanoparticles. In TB negative samples (Figure 5B), the microscope image shows dispersed brown nanoparticles. Exposure to GMNP and magnetic field did not seem to affect the staining of AFB. A study to determine the direct effect of GMNP was conducted by using the same TB positive sputum sample and subjected to NCBA and SSM. Figure 6 shows bright-field microscope images of the TB positive sample subjected to both NCBA (Figure 6A) and SSM (Figure 6B). The images show that the GMNP aggregates and concentrates the AFB when using NCBA (Figure 6A); the same sample registered negative (no visible red AFB) when using SSM (Figure 6B). Our previous study showed that NCBA increased AFB count by 47% compared to SSM [33].

Using a medical statistical software (available online: https://www.medcalc.org/calc/diagnostic_test.php, accessed on 11 December 2019), the clinical sensitivity, clinical specificity, positive predictive value, and negative predictive value were calculated and presented in Table 2. The results at 95% confidence interval (CI) for SSM showed sensitivity and specificity of 63.9% and 100.0%, respectively. The sensitivity of SSM at 64% was well within the range reported in the literature [10,13]. The clinical sensitivity and clinical specificity at 95% CI for NCBA were 100.0% and 99. 7%, respectively, compared to the standard culture. The positive predictive value (PPV) and negative predictive value (NPV) for SSM were 100.0% and 93.0%, respectively. Meanwhile, the positive predictive value and negative predictive value for NCBA were 98.5% and 100.0%, respectively. The accuracy of NCBA was 99.7%. The estimated prevalence was 17.2%.

## 4. Discussion

### 4.1. Mechanism of GMNP-Cell Interaction

GMNP binding to the Mtb bacteria is facilitated by a combination of chemical and ionic forces. In the initial stage of adhesion, the cells are brought into contact with the GMNP surface due to Brownian motion and hydrodynamic force. Next, the bacteria bind to the GMNP surface via physico-chemical and molecular interactions such as carbohydrate-protein binding and ionic charges on the cell surface, forming GMNP-cells. Furthermore, glycan is a charged molecule, thus bacterial cells that donate more electrons adhere more strongly to the surface, decreasing electrostatic repulsion. We also hypothesize that specific binding of glycan-coating on GMNP to Mtb is facilitated by cell surface antigens, such as the 45 kDa Apa (alanine- and proline-rich antigenic) and 19 kDa proteins [6,40]. Originally thought to be a secreted antigen, Ragas et al. showed that Apa can be associated with mycobacterial cell surface that is accessible to receptor moieties [40]. Apa is associated with the cell wall for a sufficiently long period of time to aid in the attachment of receptors. To date, Apa seems to be restricted to the Mtb complex strains [40]. It has also been shown that Mtb acquires iron by cell-surface sequestration [41], thus, it is also feasible for Mtb to be naturally attracted to GMNP. Furthermore, mycobacteria have an outer layer on the cell wall that appears transparent by transmission electron microscopy resulting in its being referred to as the electron transparent zone [42]. This zone consists of polysaccharides, rich in electronegative groups, and contains proteins and lipids [42]. The outer layer is predominantly composed of glycans, with glucan and arabinomannan being the predominant constituents [43]. Additionally, we also hypothesize that in the presence of a magnet, GMNP-cells acquire superparamagnetic properties (due to GMNP), causing them to move in the direction of the magnetic field and create a crowding highly enriched effect. This hypothesis is supported by the aggregation of Mtb cells as shown in the microscope slides. Figure 5 shows red-stained slides for TB positive samples (A) compared to the brown slides for the TB negative samples (B). Mtb cells on the slide are highly visible due to the concentration effect, which would likely reduce the visualization time of Mtb cells in 100 fields and minimize microscopy fatigue. Mtb cells are observed to occur in clusters, a unique property due to the action of GMNP.

### 4.2. NCBA v.s SSM vs. Culture

Results show that NCBA has the same time-to-result (T2R) as SSM but has the sensitivity of culture. On the other hand, culture takes 8 weeks to get a result. NCBA has, therefore, superior advantages over culture and SSM.

### 4.3. NCBA for TB Incidence

In this study, the aggregated TB incidence based on SSM is 110 per 100,000 population (122/1108 × 1000). This incidence rate agrees with the World Bank data of 116 per 100,000 for Peru in the year 2017 [44]. However, TB incidence varies by community as a function of so many factors, thus getting an aggregate may not provide a true picture. For example, based on Table 1, the incidence for community 5 is 190 per 100,000 (11/58 × 1000, highest among the 9 cities), while community 9 is 26 per 100,000 (5/190 × 1000, lowest among the 9 cities). On the other hand, with culture and NCBA, the incidence increased to 172 per 100,000 (191/1108 × 1000) overall. Community 5 has now a TB incidence of 362 per 100,000, while community 9 has 47 per 100,000. The constraint with culture is time (at least 8 weeks). However, with NCBA, the incidence rate calculation can be done almost in real-time, and control measures would become more specific to the community to prevent future epidemics.

### 4.4. NCBA for Detection of Early Onset of Disease

Jorgensen described the progression of TB disease where SSM could detect only in the 5th month from the start of disease infection when the patient started to feel unwell [45]. Column 2 of Table 3 shows the progression of the TB disease in a patient. In the first month, the person starts not feeling well. In the second month, the patient starts to cough during the night, with increasing intensity of coughing in the third month. In the fourth month, the patient may show a hemoptoic cough or even hemoptysis (where the patient starts to cough up blood or blood-stained mucus). In the fifth month, the patient has an onset of dyspnea or shortness of breath. Given this information, a logistic growth model (Equation 2) was used to estimate Mtb load in a patient from onset and over the 5-month period. Based on preliminary analysis, the growth rate (r) was estimated to be 0.0614/day. This growth rate matched the reported doubling rate of Mtb at 15 h [46]. The estimated carrying capacity was set to 10^5^ CFU/mL, equivalent to a smear grade of 4+ (many) [33,34] and equivalent to a bacterial load in a patient having been sick for 8 months without receiving treatment. Table 3 column 3 shows the result of the estimated Mtb load based on the logistic growth model. The table shows that Mtb count increases from an initial infection at the beginning of the month to 6 CFU/mL at the end of the first month and grows to 9 × 10^4^ CFU/mL at the end of the fifth month at which time the infection is detectable by SSM in the sputum sample, equivalent to a smear grade of 1+ (rare) or 2+ (few) [33]. Clinical observations have shown that, although bloody sputum is a sign of TB, smear microscopy does not always detect these cases, especially at early onset of hemoptysis. Given this model output and given the detection limits of NCBA and culture, NCBA and culture could be used to detect the disease as early as the second month after onset (4.0 × 10^1^ CFU/mL) as long as the sputum sample is available. While both are excellent, NCBA’s simplicity would be most useful in these cases.

### 4.5. NCBA to Profile TB Population

The results provide a glimpse of potential TB patient response. Since the detection limit of SSM is 10^4^ CFU/mL, while that of culture is reported to be 10 CFU/mL [29], the samples were classified into two groups: (1) Low bacterial load at 10^0^–10^3^ CFU/mL and (2) high bacterial load at 10^4^ CFU/mL or higher, as shown in Table 3. A sample positive in culture but negative in SSM must have a low bacterial load at 10^0^–10^3^ CFU/mL. With this data, we can infer that there were two groups of patients: (1) The proactive group, who reported to the clinic early in the stage of their symptoms while the bacterial load was still low and (2) the reactive group, who waited until very sick resulting in a high bacterial load. For example, 15% from community 1 and 8% from community 2 patients were reactive, waiting until really sick to report to the clinics. Only 2% and 3%, respectively, were proactive, reporting while the disease was early. Patients from community 5 and community 6 seemed to be more proactive. From community 5, 17% reported while the disease was early, while 19% did not report until very sick. From community 6, 11% were proactive while 18% were reactive. Patients from community 3 waited until sicker than those who reported early; patients from communities 4, 7, and 8 had even numbers of reactive and proactive groups. From community 9, 2% were proactive while 3% were reactive. NCBA could be used to provide real-time location profiles on TB and TB patient responses, which could lead to the development of proactive measures to minimize TB spread and exposure. See Table 4.

**Figure 7:**
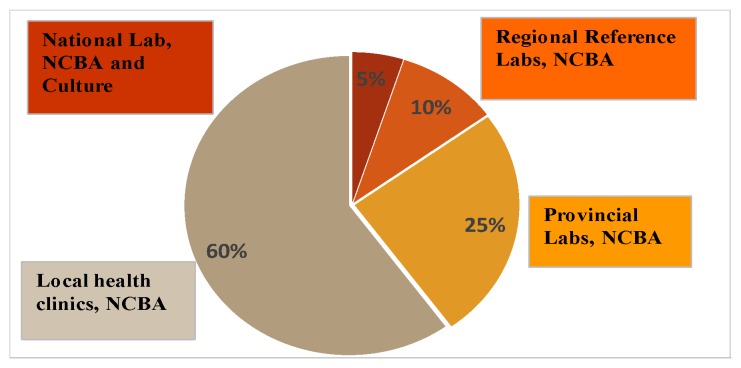
Usage of TB testing facilitated by patients and a futuristic placement of NCBA in these facilities.

Jorgensen showed that rapid tests, such as Xpert MTB/RIF and line probe assays, which are located in the National Reference Lab, were only used by 5% of patients [47]. These tests are expensive, require a fully functional lab facility and skilled personnel. However, 60% of patients reported to peripheral health clinics, which provide primary care level. Some 25% of patients were seen at health center laboratories and 10% were seen at district laboratories [47]. Figure 7 shows the distribution of the patient usage of the TB testing facilities. Figure 7 also shows a futuristic placement of NCBA in these facilities. With NCBA’s sensitivity matching that of culture, it can provide the diagnostic needs at all TB testing facilities where culture is not available.

**Figure 8:**
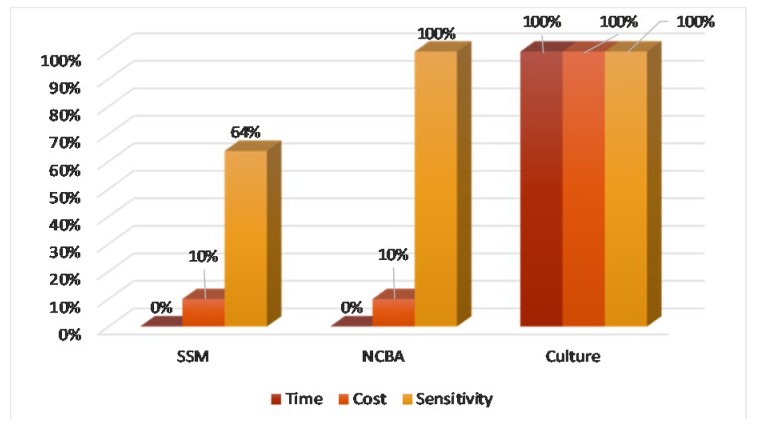
Time, cost, and sensitivity comparison of SSM, NCBA, and culture.

The most challenging aspect of TB diagnosis in rural areas is access. The three pillars of universal access are time-to-result, affordability, and sensitivity (TAS). Using culture as the gold standard, Figure 8 shows that NCBA is superior to SSM based on the TAS criteria. NCBA matches the sensitivity performance of culture at a fraction of time (20 min) and cost ($0.50/test under Peruvian condition).

## 5. Conclusions

There is a major need for providing rapid but affordable tests in local health clinics where 60%–90% of patients use and where the initial diagnosis is made [15,47]. Of the 1108 sputum samples tested, NCBA has excellent sensitivity and specificity performance matching culture at 100.0% and 99.7%, respectively. Meanwhile, SSM has 63.9% sensitivity compared to culture. Cost-wise, NCBA is comparable to SSM but much cheaper than culture. The Xpert MTB/RIF system is estimated at $20-$30/test in this region. The NCBA will also have minimal human fatigue in microscopy. NCBA could be placed in local health clinics as it only requires a microscope that is widely available in many rural areas. NCBA could detect low levels of bacterial load comparable to culture. Early detection can lead to early treatment, while the patient’s immune system is still high. Recovery is shown to be faster for patients with high immunity. The gain in time is critical as TB is airborne and highly infectious, minimizing contact exposure.

## Figures and Tables

**Table 1:** Distribution of samples and results by SSM, NCBA, and culture by city.

City #	No. Samples		SSM		NCBA		Culture	
	No.	Fraction	Positive	Negative	Positive	Negative	Positive	Negative
1	98	9%	15	83	18	80	17	81
2	119	11%	9	110	13	106	13	106
3	213	19%	36	177	50	163	50	163
4	72	6%	6	66	11	61	11	61
5	58	5%	11	47	21	37	21	37
6	123	11%	22	101	36	87	36	87
7	82	7%	5	77	10	72	9	73
8	153	14%	13	140	25	128	25	128
9	190	17%	5	185	10	180	9	181
Total	1108	100%	122	986	194	914	191	917

**Table 2:** Statistical analysis of SSM and NCBA at a 95% confidence interval (CI).

	Sensitivity	Specificity	PPV	NPV	Accuracy
**SSM**	63.9% (56.6–70.7)	100.0% (99.6–100.0)	100.0%	93.0% (91.7–94.1)	93.8% (92.2–95.1)
**NCBA**	100.0% (98.1–100.0)	99.7% (99.1–99.9)	98.5% (95.4–99.5)	100.0%	99.7% (99.2–99.9)

**Table 3:** Progression of TB disease and sensitivity of detection techniques.

End of Month	Symptoms	Est. Mtb Load in Patient, CFU/mL	Detectable by SSM and Est. Grade?	Detectable by NCBA?	Detectable by Culture?
1st	Not feeling well	6.3 x10^0^			
2nd	Cough at night	4.0 × 10^1^	No	Yes	Yes, result in 4th month
3rd	Intense coughing	2.5 × 10^2^	No	Yes	Yes, result in 5th month
4th	Onset of hemoptoic cough or hemoptysis	1.6 × 10^3^	No	Yes	Yes, result in 6th month
5th	Onset of dyspnea	9.1 × 10^3^	Yes, 1+ and 2+	Yes	Yes, result in 7th month
6th	Dyspnea	3.9 × 10^4^	Yes, 3+	Yes	Yes, result in 8th month
7th	Dyspnea	8.0 × 10^4^	Yes, 4+	Yes	Yes, result in 9th month

Smear Grade: 1+ = rare; 2+ = few; 3+ = moderate; 4+ = many.

**Table 4:** Positive samples grouped into low and high bacterial load, CFU/mL.

City	No. Samples	SSM+	NCBA+		Culture+	
		>10^4^ (High)	10^1–^10^3^ (Low)	>10^4^ (High)	10^1–^10^3^ (Low)	>10^4^ (High)
1	98	15%	3%	15%	2%	15%
2	119	8%	3%	8%	3%	8%
3	213	17%	7%	17%	7%	17%
4	72	8%	7%	8%	7%	8%
5	58	19%	17%	19%	17%	19%
6	123	18%	11%	18%	11%	18%
7	82	6%	6%	6%	5%	6%
8	153	8%	8%	8%	8%	8%
9	190	3%	3%	2%	2%	3%
Total	1108	11%	6%	11%	6%	11%
